# Accuracy Comparison between Robot-Assisted Dental Implant Placement and Static/Dynamic Computer-Assisted Implant Surgery: A Systematic Review and Meta-Analysis of In Vitro Studies

**DOI:** 10.3390/medicina60010011

**Published:** 2023-12-20

**Authors:** Saurabh Jain, Mohammed E. Sayed, Wael I. Ibraheem, Abrar A. Ageeli, Sumir Gandhi, Hossam F. Jokhadar, Saad Saleh AlResayes, Hatem Alqarni, Abdullah Hasan Alshehri, Halah Mohammed Huthan, Atheer Alami, Mohammed Hussain Dafer Al Wadei, Yahya Aljabri

**Affiliations:** 1 Department of Prosthetic Dental Sciences, College of Dentistry, Jazan University, Jazan 45142, Saudi Arabia; 2 Department of Preventive Dental Sciences, College of Dentistry, Jazan University, Jazan 45142, Saudi Arabia; wibraheem@jazanu.edu.sa; 3 College of Dentistry, Jazan University, Jazan 45142, Saudi Arabia; abrarageeli@gmail.com (A.A.A.); r.h.r-053@hotmail.com (H.M.H.); 201803597@stu.jazanu.edu.sa (A.A.); yahya.2000.sulaiman@gmail.com (Y.A.); 4 Dental Public Health, The University of Sheffield, Sheffield S10 2TN, UK; 5 NYU College of Dentistry, New York, NY 10010, USA; sumirgandhi@gmail.com; 6 Department of Oral and Maxillofacial Prosthodontics, Faculty of Dentistry, King Abdulaziz University, Jeddah 21589, Saudi Arabia; hjokhadar@kau.edu.sa; 7 Department of Prosthetic Dental Sciences, College of Dentistry, King Saud University, Riyadh 11545, Saudi Arabia; salresayes@ksu.edu.sa; 8 Department of Prosthetic Dental Sciences, College of Dentistry, King Saud Bin Abdulaziz University for Health Sciences, Riyadh 14611, Saudi Arabia; qarnih@ksau-hs.edu.sa; 9 King Abdullah International Medical Research Center, Riyadh 14611, Saudi Arabia; 10 Department of Prosthodontics, Faculty of Dentistry, King Khalid University, Abha 62527, Saudi Arabia; abhalshehri@kku.edu.sa; 11 Department of Restorative Dentistry, Faculty of Dentistry, King Khalid University, Abha 62527, Saudi Arabia; moalwadai@kku.edu.sa

**Keywords:** dental implants, accuracy, computer-assisted implant surgery, robot-assisted implant surgery, navigation systems, surgical robot

## Abstract

*Background and Objectives*: The present systematic review and meta-analysis undertake a comparison of studies that examine the accuracy of robot-assisted dental implant placement in relation to static computer-assisted implant surgery (SCAIS), dynamic computer-assisted implant surgery (DCAIS), and freehand procedures. This study aims to provide a comprehensive understanding of the precision of robot-assisted dental implant placement and its comparative efficacy in relation to other placement techniques. *Methods*: The guidelines recommended by Preferred Reporting Items for Systematic Reviews and Meta-Analyses (PRISMA) were used to organize and compose this review. Four electronic databases (PubMed, Web of Science, Scopus, and Cochrane) were systematically searched for pertinent articles. Articles were selected following the inclusion and exclusion criteria. Qualitative and quantitative analyses of the selected articles were performed. *Results*: The initial electronic search resulted in 1087 hits. Based on the inclusion and exclusion criteria, five articles were selected for qualitative analysis, out of which three were considered for quantitative analysis. Three parameters were considered for accuracy evaluation (angular, coronal, and apical deviation). The mean angular deviation was −1.22 degrees (95% CI, −1.06–−1.39), the mean coronal deviation was −0.15 mm (95% CI, −0.24–−0.07), and the mean apical deviation was −0.19 mm (95% CI, −0.27–−0.10). *Conclusions*: The robotic implant system was found to have significantly lower angular deviations and insignificantly lower coronal and apical deviations compared to DCAIS. Within the limitations of this review, it can be concluded that robot-assisted implant placement in resin models permits higher accuracy compared to DCAIS and SCAIS systems. However, due to the limited number of comparative studies with high heterogeneity, the findings of this review should be interpreted with caution. Further research is necessary to confirm the clinical application of robotics in implant surgery.

## 1. Introduction

Dental implants are commonly and widely used for the oral rehabilitation of partially and completely edentulous patients [1], with other specific applications in maxillofacial prosthodontics and orthodontics [2]. Due to improved modern understanding of biomechanical aspects that primarily encompass the influence of load distribution on the biochemistry and physiology at the implant–bone interface [3], diagnostic and planning technologies [4], surgical techniques [5,6], and loading protocols [7,8], a high level of predictability can be achieved with dental implants to attain favorable treatment outcomes. For an implant to be stable in the long term and esthetically pleasing, it is imperative that it should be placed accurately in the correct three-dimensional position, with proper angulation [9] and optimum depth with respect to the surrounding bone and adjacent/opposing teeth [10]. Traditional freehand osteotomy preparation for conventional dental implants has been centered on two-dimensional or three-dimensional radiographic images. The success of this procedure is subject to the dentist’s clinical experience [11] and the subsequent ability to place implants accurately [12]. Subjective interpretation errors can jeopardize the longevity of implants or may lead to compromised esthetic outcomes. Another primary concern with improper osteotomy is the damage inflicted on critical anatomical structures, which may lead to long-term complications [13,14]. The use of advanced planning software and prototyping techniques has facilitated the introduction of the concept of computer-assisted implant surgery (CAIS), which includes digital pre-surgical planning and guided surgical procedures [10,15]. CAIS is classified into two types: (a) static computer-assisted implant surgery (SCAIS) and (b) dynamic computer-assisted implant surgery (DCAIS) [16,17,18,19]. In SCAIS, a template guides the dentist in achieving proper angulation and depth of the implant osteotomy site, thus aiding in improving the long-term treatment prognosis [16,18]. The limitations of SCAIS include additional time for surgical guide fabrication, restricted view of the surgical site, inadequate cooling of the surgical site, failure to incorporate dynamic changes in the implant positioning, and incorporation of technical errors [17,20]. DCAIS involves using dynamic navigation systems during the surgical phase of implant treatment [17,21]. This system increases the accuracy of implant placement and prevents damage to critical anatomical structures like the inferior alveolar nerve, the maxillary sinus, etc. [22]. With DCAIS, the dentist can make dynamic changes in implant positioning during surgery. Most disadvantages of SCAIS can be overcome using this system [17,22].

Various systematic reviews have found high implant placement accuracy using DCAIS, compared to SCAIS and freehand implant surgeries [23,24,25]. Schnutenhaus et al. [25], in their systematic review and meta-analysis, reported that studies using DCAIS resulted in angular, coronal, and static deviation values of 4.1 degrees (in vitro) and 3.7 degrees (clinical), 1.03 mm (in vitro) and 1 mm (clinical), and 1.04 mm (in vitro) and 1.33 mm (clinical), respectively. However, for SCAIS, the mean angular, coronal, and apical deviation values were 3.6 degrees, 1.1 mm, and 1.40 mm, respectively. The values for freehand surgery were reported to be very high (9.9 degrees, 2.77 mm, and 2.91 mm). They reported comparable clinical acceptability of DCAIS with SCAIS. Jorba-García et al. [24] reported that DCAIS is more accurate than SCAIS and freehand surgery. They reported that studies using DCAIS resulted in angular, coronal, and apical deviation values of 2.84 degrees, 0.75 mm, and 1.049 mm, respectively. Bover-Ramos et al. [23] also reported that fully guided surgery is more accurate than half-guided surgery. They reported that the studies using DCAIS resulted in angular, coronal, and apical deviation values of 3.13 degrees, 1 mm, and 1.91 mm, respectively, whereas the values reported by studies using SCAIS were 4.30 degrees, 1.4 mm, and 1.23 mm, respectively.

Studies have also reported that the use of DCAIS reduces the concentration levels of the dentist during the surgical phase due to the constant maneuvering of focus between the osteotomy site and dynamic navigation displayed on the screen [20,26]. This system requires a mandatory training period [27], and the outcome depends on the expertise [28] and proficiency of the dentist [29]. Successful application of robots in sensitive specialized surgical treatments (like pediatric neurosurgery) [30] has led to their introduction in dental treatments [31], including the more complex zygomatic implant placement [32]. Surgical robots have been found to be competitive and efficient in terms of their position feedback [32], and comparatively accurate with digital surgical guides [33]. They have also been reported to have a high degree of autonomy [34]. Such an advanced feature permits a robot to adjust during the surgical procedure, thereby executing surgical tasks without being controlled by the surgeon [34]. The robot-assisted implant system consists of two main parts: (a) a mechanical robotic arm, operated by the software, which holds the handpiece and performs the surgical part with the help of the operating software; and (b) a tracking device that uses light to monitor and guide the process [21,31,35,36,37,38].

The first commercially available FDA-approved robot for dental implant surgery was developed in 2017 and was named YOMI. It was a semi-active robot system capable of performing autonomous drilling and implant placement [35]. With time, fully automatic robotic systems were introduced [36], which could also perform pre-surgical treatment planning along with autonomous drilling, and implant placement using a tracking/guiding mechanism [37]. Robotic systems can reduce dentist-related errors (like fatigue) and have been shown to provide higher accuracy for implant placement [21]. However, the dentist can observe the surgical phase and control the procedure in case of any complications [21,32,33].

Several studies published in the literature comparing the accuracy (angular deviation, coronal deviation, and apical deviation) of dental implants placed using a new generation of surgical robots with other surgical techniques (freehand, SCAIS, and DCAIS) have reported varied outcomes [13,21,31,38,39]. The results of these studies are relevant for dentists and manufacturing companies, as they can guide the selection and improvement of new-generation robotic surgical systems. The current literature lacks a systematic review that evaluates the accuracy of dental implants placed using robotic systems compared to other techniques like freehand surgery, SCAIS, and DCAIS. Thus, this review aims to compare and analyze the studies comparing the accuracy of dental implants placed using robotic systems compared to those placed using freehand surgery, SCAIS, and DCAIS. The tested null hypothesis is that there is no difference in the accuracy of dental implants placed using robotic systems compared to those placed using freehand surgery, SCAIS, and DCAIS.

## 2. Materials and Methods

### 2.1. Permission and Registration

The Preferred Reporting Items for Systematic Reviews and Meta-Analyses (PRISMA) specifications were used to organize and compose the current systematic review [40]. The protocol of the current review was registered with the International Prospective Register of Systematic Reviews (Prospero registration no: CRD42023451704).

### 2.2. Selection Criteria

The details of the inclusion and exclusion criteria are listed in Table 1.

### 2.3. Exposure and Outcome

The exposure in the present study was the surgical placement of dental implants using a robotic system and freehand surgery/DCAIS/SCAIS. The outcome was the accuracy of the placement in terms of angular, coronal, and apical deviations. The focused PICO/PECO (population, intervention, comparison, outcome/population, exposure, comparison, outcome) question for the current study was ‘Does robotic system-assisted dental implant site preparation or implant placement (intervention/experiment) in partially or completely edentulous resin models/phantom (participant/population) have the same accuracy (outcome) compared to dental implant site preparation/implant placement assisted by freehand surgery or DCAIS or SCAIS (comparator)?’

### 2.4. Search Strategy, Study Selection, and Data Extraction

We independently performed an electronic search of the available literature on four databases using the following search strings: ‘dental implants’ AND ‘dental implant robotic system’ AND ‘computer-assisted surgery’ OR ‘computer-guided implantology’ AND accuracy. Truncation and Boolean operators were used. Minor changes were made according to each electronic database (Table 2). 

The primary medical databases searched were PubMed, Scopus, Web of Sciences, and Cochrane. The search was conducted in August 2023, and the search was limited to articles in the English language that were published in the last ten years. Duplicate articles were removed, and there was no discrepancy between the articles. Two independent reviewers read the titles and abstracts of the collected articles and selected the relevant articles based on pre-determined selection criteria. References from these articles were searched manually for relevant articles. Grey literature was also searched for relevant articles, but no new titles were detected. Later, two different reviewers independently reviewed their full texts and shortlisted the relevant articles for final consideration. Any differences between the reviewers were resolved by consensus between the three authors. 

A self-designed Performa was used to extract relevant data from the selected articles (Table 3 and Table 4). Table 3 provides information related to the primary author’s name, year and country of publication, number of resin models and implants placed, type of implant system and their dimensions, details about planning and navigation software, details related to a surgical robotic system and type of comparator surgical approach used for implant placement. Table 4 details the outcomes of the accuracy parameters tested in each selected study, which included angular, coronal, and apical deviations, and summarizes the conclusions of these studies.

### 2.5. Quality Assessment

Quality analysis of all the selected articles was accomplished using the Modified CONSORT scale for in vitro studies [41]. Details about all fourteen items included are mentioned in Table 5. 

### 2.6. Quantitative Assessment

Data extraction was performed by two authors individually. Three studies provided data and were used for a meta-analysis. The meta-analysis was performed using Review Manager 5.4.1 [42]. All studies provided data for three parameters: angular deviation, apical deviation, and coronal deviation. All parameters are continuous; therefore, the inverse variance was used. Since they are exact measurements, a fixed-effect model was used. Heterogeneity was analyzed using the I2 test. The overall effect was computed as the mean difference in divisions between the groups, and *p* < 0.05 was considered a statistically significant difference.

## 3. Results

### 3.1. Identification and Screening

**Figure 1:**
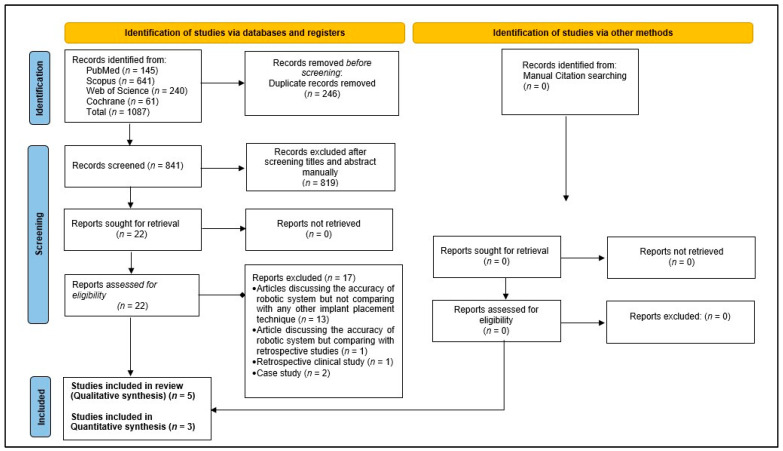
Flow chart of the study selection process in accordance with the Preferred Reporting Items for Systematic Review and Meta-Analysis.

The initial electronic search resulted in 1087 hits, out of which 246 titles were duplicates and were removed. After screening the titles and abstracts, 819 were found to be irrelevant (based on the article selection criteria) and excluded. The full manuscript of the selected 22 titles was read, and their references were explored for any additional relevant titles, but no relevant titles were discovered. Thirteen of the selected twenty titles were excluded, as those studies only measured the accuracy of robot-assisted implant surgery but did not compare it to any other surgical techniques; one study compared the accuracy of robot-assisted implant surgery with previously published studies, two were case reports, and one was a retrospective clinical study. Finally, five articles were selected for qualitative analysis, of which three were considered for a meta-analysis (Figure 1). 

### 3.2. Quality Assessment

Out of the total 75 reported entries, 50 (66.66%) were positive (Table 5). A study by Chen et al. [38] reported the greatest score (13 out of 15), whereas a study by Cao et al. [31] reported the lowest score (6 out of 15). None of the included studies reported details on the measures taken to conceal the random allocation and its implementation. Only one study [38] reported the mode of randomization. Two studies [13,38] provided details related to sample size determination and accessibility of the complete data. Three studies [13,21,38] provided details of blinding, whereas comparisons of outcomes were statistically compared in four studies [13,21,38,39]. All selected studies adequately reported details related to the introduction, intervention, outcomes, study limitations, and funding sources. 

### 3.3. Features of the Selected Studies

All five included studies were published in the last four years (2020–2023). Most of these studies (four out of five) were from China [13,21,31,38] and one was from Korea [39]. Four studies compared the accuracy of robot-assisted implant surgeries with DCAIS [13,21,31,38], whereas one study compared the accuracy of robot-assisted implant surgeries with SCAIS [39]. A total of 610 implants were placed (306 by robotic systems and 304 by computer-assisted surgeries). In one study, implants were placed in maxillary [38] and mandibular [39] resin models. In two other studies [13,21], both maxillary and mandibular resin models were used, whereas zygomatic implants were placed for accuracy assessment in one study [31]. All the studies used different robotic systems for implant placement. Three studies provided details about the type of planning software and navigation system used for treatment planning and guided implant placement, all of which were different. Cone beam computed tomography (CBCT) was performed for each study for treatment planning and accuracy evaluation post-operatively (along with accuracy evaluation software). The implant system used in each study was different. As per the predefined inclusion criteria, for a study to be included, it should report at least these three parameters: angular deviation, coronal/entry/platform deviation, and apical/exit/apex deviation. Some of the included studies reported various other parameters, too. Therefore, Forest plots were drawn for these three parameters only.

#### 3.3.1. Angular Deviation

**Figure 2:**
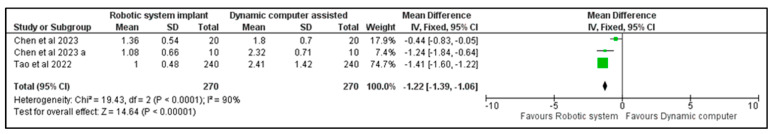
Forest plot comparing the angular deviation values of the robot-assisted implant with DCAIS [13,21,38].

All five studies reported higher angular deviations for CAIS compared to robotic systems. The robotic system reported a mean angular deviation of 2.38 ± 0.62 degrees compared to 3.16 ± 3.36 with SCAIS [39]. For zygomatic implants, an angular deviation of 1.52 ± 0.58 degrees was reported by the robotic system compared to 2.07 ± 0.30 with DCAIS [31]. Chen et al. [38] reported lower angular deviations of 1.94 ± 0.66 degrees when implants were placed in fresh extraction sites with the robotic system compared to DCAIS (3.44 ± 1.38). Three studies provided mean and standard deviation data and were included in the meta-analysis [13,21,38]. Although a statistically significant heterogeneity was present between these studies (I2 = 90%, *p* < 0.0001), they all favored robotic system implants. The mean angular deviation was 1.22 degrees lower in Robotic System Implants (95% CI, −1.06–−1.39). Robotic system implants showed significantly lower angular deviations than DCAIS, with *p* < 0.00001 (Figure 2).

#### 3.3.2. Coronal Deviation

**Figure 3:**
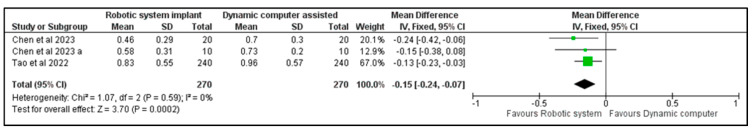
Forest plot comparing the coronal deviation values of the robot-assisted implant with DCAIS [13,21,38].

Four studies reported higher coronal deviations for DCAIS compared to robotic systems [13,21,31,38], whereas Jin et al. [39] reported a higher coronal deviation of 0.61 ± 0.29 mm with the robotic system compared to a 0.49 ± 0.39 with SCAIS. For zygomatic implants, a coronal deviation of 0.79 ± 0.19 mm was reported with the robotic system compared to 0.96 ± 0.28 with DCAIS [31]. Chen et al. [38] reported higher coronal deviations of 0.86 ± 0.36 mm when implants were placed in fresh extraction sites with the robotic system compared to DCAIS (0.70 ± 0.21). The same three studies provided data for coronal deviations, and a meta-analysis was performed [13,21,38]. The studies showed no heterogeneity, with all studies favoring robotic system implants (I2 = 0%, *p* = 0.39). The mean coronal deviation was 0.15 mm lower in the robotic system implant (95% CI, −0.24 to −0.07). Robotic system implants showed significantly lower coronal deviation than DCAIS, which was statistically significant (Figure 3).

#### 3.3.3. Apical Deviation

**Figure 4:**
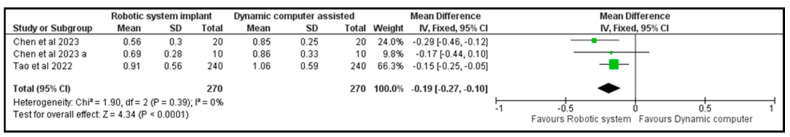
Forest plot comparing the apical deviation values of the robot-assisted implant with DCAIS [13,21,38].

All five studies reported higher apical deviations for CAIS compared to robotic systems. A coronal deviation of 0.50 ± 0.14 mm was reported with the robotic system compared to 0.72 ± 0.34 with SCAIS [39]. For zygomatic implants, an apical deviation of 1.49 ± 0.48 mm was reported with the robotic system compared to 2.26 ± 0.32 with DCAIS [31]. Chen et al. [38] reported lower apical deviations of 0.77 ± 0.34 mm when implants were placed in fresh extraction sites with the robotic system as compared to DCAIS (0.95 ± 0.38). Data were also available for apical deviation from the three studies. The studies depicted similar patterns with no heterogeneity (I2 = 0%, *p* = 0.39). The mean apical deviation was 0.19 mm (95% CI, −0.27–−0.10) lower in the robotic implant system than DCAIS. This difference was statistically significant, with *p* < 0.00001 (Figure 4).

## 4. Discussion

The introduction of robot-assisted implant surgery has facilitated the accurate placement of dental implants as per the treatment plan. The present study is the first review that analyzed and summarized all published in vitro studies comparing the accuracy of robot-assisted dental implants to implants placed via freehand surgery, SCAIS, or DCAIS. General outcomes revealed that the type of surgical approach affects the accuracy of implant placement. Thus, the tested null hypothesis can be rejected. The accuracy measurements varied in each study and with the type of accuracy parameter measured. The cause of this heterogeneity can be attributed to the fact that all the included studies used different types of robotic and computer-assisted systems for implant placement. The planning and navigation software was also different, and there were differences in the placement location and the implant company used. This systematic review was limited to in vitro studies as there are not enough clinical studies comparing the accuracy of robotic systems with other implant placement systems. A clinical study by Jia et al. [43] compared robot-assisted and static navigation-assisted implants. They reported a significantly higher accuracy for the robot-assisted implant (angular deviation: 1.48 ± 0.59 degrees, coronal deviation: 0.43 ± 0.18 mm, and apical deviation: 0.56 ± 0.18 mm) compared to the static navigation-assisted implant (angular deviation: 2.42 ± 1.55 degrees, coronal deviation: 1.31 ± 0.62 mm, and apical deviation: 1.47 ± 0.65 mm). Mozer et al. [44], in their case report, reported angular deviations between 0 and 1 degree (coronal, apical, and vertical deviations of 0.3–0.5 mm, 0.5 mm, and 0.4 mm, respectively). They concluded that deviations in robot-assisted implant surgeries are comparable to static navigation-assisted surgeries. A similar clinical study by Chen et al. [45] reported higher accuracy for implants (angular, coronal, and apical deviations of 2.81  ±  1.13°, 0.53  ±  0.23 mm, 0.53  ±  0.24 mm, respectively) placed via robot-assisted surgery compared to DCAIS and SCAIS. Bolding and Reebye [35] reported angular, coronal, and apical deviations of 2.56  ±  1.48°, 1.04  ±  0.70 mm, 0.95  ±  0.73 mm, respectively, in completely edentulous patients using robot-assisted surgeries. 

Chen et al. [38] reported higher deviations (angular, coronal, and apical) in implants placed in models replicating fresh extraction sites compared to healed sites, which were reported to be related to the anatomical features of the extraction sites. Lower angular deviations were reported in fresh extraction sites assisted in robotic surgery (1.94 ± 0.66 mm) compared to DCAIS (3.44 ± 1.38), which was attributed to the better stability of the robotic arm that could maintain the orientation of the surgical drills. The coronal deviation was reported to be higher for the robotic system (0.86 ± 0.36) compared to DCAIS (0.70 ± 0.21), which was due to the minor sliding movement of the robotic arm along the bone wall in an attempt to control the drilling direction during surgical site preparation [46]. In three out of five studies [13,38,39], partially edentulous arches were used to compare the accuracy of implant placement. One study used completely edentulous arches [31], whereas another study used both partially and completely edentulous arches [21]. The difficulties of dental implant surgery in dentulous or partially edentulous arches may vary, which may affect the accuracy. Most of the included studies reported higher accuracy of implants placed with robotic systems compared to other techniques. In a study by Tao et al. [21], implants were placed in both edentulous and partially edentulous phantoms. They reported almost similar values for angular, coronal, and apical deviations for both edentulous and partially edentulous situations (Table 4). They also reported lower angular deviations in mandible phantoms in robotic system implants compared to DCAIS. 

The surgical robot reduces errors caused by operator fatigue, tremors, compromised posture, and blind spots during surgical site preparation [13], whereas robots have a stiff, stable mechanical surgical arm that is operated by a tracking software, thus increasing the accuracy. Robot-assisted implant surgeries do not require dentists to have high surgical skills other than training to control the robot operation accurately [47]. Studies have reported that a few factors may cause implant placement inaccuracies during robotic surgeries. Chen et al. [38] reported that differences in the density of buccal and palatal bone might lead to the sliding of the robotic arm and increased apical deviations in robot-assisted implant surgeries in freshly extracted sockets. In addition, visible light positioning technology used by robots to track position may be influenced by the ambient light, leading to inaccuracies [38]. All the selected studies were laboratory-based studies performed on resin models. These cannot simulate real oral clinical environments, which involve the presence of oral fluids and blood, restricted mouth opening, the head position of patients, possibilities of patient and tongue movement, and differences in bone density [13,21,38]. The use of robots in dental implantology is still restricted due to their limited intelligence, higher cost of machine and software, complex structure and workflow, and the large volume of the machine [13,21,28,38,48]. With rapid advancements in technology, robots are expected to be cost-effective; advanced software and the cost-effectiveness of robots may lead to their widespread clinical use in dental implantology.

### Strength and Limitations

The highlights of the present review are its comprehensive article search plan, unbiased independent article review, and selection of the concerned authors. All published articles related to robotic implant placement were reviewed to avoid the loss of pertinent articles. The present review proposes that more clinical comparative studies with higher sample sizes be conducted in the future to improve the treatment outcome and longevity of dental implants and minimize complications. The present systematic review is limited by the fact that only in vitro studies were included, which may not simulate actual oral conditions. The number of studies included is limited, with low-to-high quality and a lack of homogeneity. In addition, the difficulty levels of implant placement may vary with the site of placement and edentulous state (partial or complete edentulous). Few of the included studies were pilot studies. Only three articles were considered in the meta-analysis due to heterogeneity. More studies with consistency in the surgical approach and a higher sample size are required to provide decisive conclusions.

## 5. Conclusions

Within the limitations of this review, it can be concluded that robotic-assisted implant placement in resin models permits higher accuracy compared to DCAIS and SCAIS systems. However, due to the limited number of comparative studies with high heterogeneity, the findings of this review should be interpreted with caution. Further research is necessary to confirm the clinical application of robotics in implant surgery. 

## Figures and Tables

**Table 1:** Inclusion and exclusion criteria for selecting the research articles.

Issue	Inclusion	Exclusion
Language	English	Non-English
Publication type	Peer-reviewed academic journals	Non-indexed journal publications
Study Design	In vitro studies in which implants were placed on edentulous or partially edentulous resin jaw models	Case reports, case series, clinical studies, electronic posters, animal studies, reviews, cadaver studies, and non-peer-reviewed reports
Availability	Available online (abstract and full text) and/or print on bibliographic databases	Not available on bibliographic databases
Time period	January 2013 to July 2023	Prior to December 2012
Articles included and data extraction	Agreement between two independently working authors	Disagreement between the two independently working authors
Sample size	Studies in which more than ten implants were placed using robotic surgery (including pilot studies); power analysis was performed, and the sample size requirements were met	Studies that evaluated the accuracy of robotically placed implants but did not compare them to the accuracy of implants placed via static/dynamic navigation, or the freehand technique
Relevance/conditions	Studies that compared the accuracy of implants placed with the help of robotics to those placed via other techniques (static navigation and dynamic navigation of freehand techniques)	Studies that evaluated orthodontic retention mini-implants and pterygoid implants
Outcome parameters	Studies that documented and compared the following minimal parameters: coronal, apical, and angular deviation	Studies that compared the accuracy of implants placed via techniques other than robotics (static navigation and dynamic navigation of freehand technique)

**Table 2:** Search strings and strategy.

Database	Combination of Search Terms and Strategy	Number of Titles
MEDLINE-PubMed	((“dental implants”[MeSH Terms] OR “dental implantation”[MeSH Terms] OR “resin models”[Title/Abstract] OR “printing, three dimensional”[MeSH Terms] OR (“3-D”[All Fields] AND “printed model”[Title/Abstract]) OR “jaw, edentulous”[MeSH Terms] OR “zygomatic implant *”[Title/Abstract] OR “immediate implant placement”[Title/Abstract] OR “implantology”[Title/Abstract] OR “jaw, edentulous, partially”[MeSH Terms] OR “phantom study”[Title/Abstract] OR “implant drilled socket”[Title/Abstract] OR “in vitro techniques”[MeSH Terms]) AND (“robotics”[MeSH Terms] OR “robot*”[Title/Abstract] OR “robotic surgical procedures”[MeSH Terms] OR “robot technology”[Title/Abstract] OR “robotic surgery”[Title/Abstract] OR “dental implant robotic system”[Title/Abstract] OR “robot assisted surgery”[Title/Abstract] OR “surgical robot”[Title/Abstract] OR “robot assisted”[Title/Abstract] OR “implant surgery”[Title/Abstract] OR “robot assisted dental implant surgery”[Title/Abstract] OR “Yomi”[Title/Abstract]) AND (“dynamic navigation”[Title/Abstract] OR “static navigation”[Title/Abstract] OR “computer aided surgery”[Title/Abstract] OR “computer assisted surgery”[Title/Abstract] OR “computer guided implantology”[Title/Abstract] OR “computer guided surgery”[Title/Abstract] OR “computer aided dental implant”[Title/Abstract] OR “navigation systems”[Title/Abstract] OR “dynamic computer assisted surgery”[Title/Abstract] OR “static computer assisted surgery”[Title/Abstract] OR “freehand surgery”[Title/Abstract] OR “freehand drilling”[Title/Abstract] OR “dynamic navigation system”[Title/Abstract] OR “surgical navigation”[Title/Abstract] OR “static guided implant surgery”[Title/Abstract] OR “dental navigation”[Title/Abstract] OR (((“three”[All Fields] OR “threes”[All Fields]) AND (“dimensional”[All Fields] OR “dimensionalities”[All Fields] OR “dimensionality”[All Fields] OR “dimensionalized”[All Fields] OR “dimensionally”[All Fields])) AND “dental planning”[Title/Abstract]) OR (“3D”[All Fields] AND “dental planning”[Title/Abstract]) OR “surgical guide”[Title/Abstract] OR “surgical stent”[Title/Abstract] OR “surgical template”[Title/Abstract]) AND (“accuracy”[Title/Abstract] OR “dimensional measurement accuracy”[MeSH Terms] OR (“computer guided”[All Fields] AND “accuracy”[Title/Abstract]) OR (“computer guided”[All Fields] AND “precision”[Title/Abstract]))) AND ((y_10[Filter]) AND (english[Filter]))	145
Scopus	(“dental implants” OR “dental implantation” OR “resin models” OR “resin models” OR “printing, three dimensional” OR “3-D” AND “printed model” OR “jaw, edentulous” OR “zygomatic implant*” OR “immediate implant placement” OR “implantology” OR “jaw, edentulous, partially” OR “phantom study” OR “implant drilled socket” OR “in vitro techniques”) AND (“robotics” OR “robot*” OR “robotic surgical procedures” OR “robot technology” OR “robotic surgery” OR “dental implant robotic system” OR “robot assisted surgery” OR “surgical robot” OR “robot assisted” OR “implant surgery” OR “robot assisted dental implant surgery” OR “Yomi”) AND (“dynamic navigation” OR “static navigation” OR “computer aided surgery” OR “computer assisted surgery” OR “computer guided implantology” OR “computer guided surgery” OR “computer aided dental implant” OR “navigation systems” OR “dynamic computer assisted surgery” OR “static computer assisted surgery” OR “freehand surgery” OR “freehand drilling” OR “dynamic navigation system” OR “surgical navigation” OR “static guided implant surgery” OR “dental navigation” OR “ three dimensional dental planning” OR “3D dental planning” OR “surgical guide” OR “surgical stent” OR “surgical template”) AND (“accuracy” OR “dimensional measurement accuracy” OR “computer guided accuracy” OR “computer guided precision”) AND PUBYEAR > 2013 AND PUBYEAR < 2023 AND (LIMIT-TO (SUBJAREA, “DENT”)) AND (LIMIT-TO (DOCTYPE, “ar”)) AND (LIMIT-TO (LANGUAGE, “English”)) AND (LIMIT-TO (SRCTYPE, “j”))	641
Web of Sciences (Core collection)	#1 (P) TS = (“dental implants” OR “dental implantation” OR “resin models” OR “resin models” OR “printing, three dimensional” OR “3-D” AND “printed model” OR “jaw, edentulous” OR “zygomatic implant*” OR “immediate implant placement” OR “implantology” OR “jaw, edentulous, partially” OR “phantom study” OR “implant drilled socket” OR “in vitro techniques”) and English (Languages) #2 (I) TS = (“robotics” OR “robot*” OR “robotic surgical procedures” OR “robot technology” OR “robotic surgery” OR “dental implant robotic system” OR “robot assisted surgery” OR “surgical robot” OR “robot assisted” OR “implant surgery” OR “robot assisted dental implant surgery” OR “Yomi” ) and English (Languages) #3 (C) TS = (“dynamic navigation” OR “static navigation” OR “computer aided surgery” OR “computer assisted surgery” OR “computer guided implantology” OR “computer guided surgery” OR “computer aided dental implant” OR “navigation systems” OR “dynamic computer assisted surgery” OR “static computer assisted surgery” OR “freehand surgery” OR “freehand drilling” OR “dynamic navigation system” OR “surgical navigation” OR “static guided implant surgery” OR “dental navigation” OR “ three dimensional dental planning” OR “3D dental planning” OR “surgical guide” OR “surgical stent” OR “surgical template”) and English (Languages) #4 (O) TS = (“accuracy” OR “dimensional measurement accuracy” OR “computer guided accuracy” OR “computer guided precision”) and English (Languages) #4 AND #3 AND #2 AND #1 Indexes = SCI-EXPANDED, SSCI, A&HCI, CPCI-S, CPCI-SSH, ESCI, CCR-EXPANDED, IC Timespan = Timespan: 1 January 2013 to 1 August 2023 (Publication Date) and English (Languages)	240
Cochrane Library	#1 MeSH descriptor: [Dental Implants] explode all trees #2 MeSH descriptor: [Dental Implants] explode all trees #3 MeSH descriptor: [Jaw, Edentulous] explode all trees #4 MeSH descriptor: [Printing, Three-Dimensional] explode all trees #5 MeSH descriptor: [In Vitro Techniques] explode all trees #6 MeSH descriptor: [Jaw, Edentulous, Partially] explode all trees #7 resin models #8 immediate implant placement #9 implantology #10 Zygomatic Implant* #11 Phantom study #12 implant drilled socket #13 3 D Printed Model #14 MeSH descriptor: [Robotics] explode all trees #15 MeSH descriptor: [Robotic Surgical Procedures] explode all trees #16 robot * #17 robot technology #18 robotic surgery #19 dental implant robotic system #20 robot-assisted surgery #21 surgical robot #22 Robot-assisted #23 implant surgery #24 robot assisted dental implant surgery #25 Yomi #26 dynamic navigation #27 Static navigation #28 computer-aided surgery #29 computer-assisted surgery #30 Computer-guided implantology #31 computer-guided surgery #32 computer aided dental implant #33 Navigation systems #34 Dynamic computer-assisted surgery #35 static computer-assisted surgery #36 Freehand surgery #37 Freehand drilling #38 dynamic navigation system #39 surgical navigation #40 Static-guided implant surgery #41 Dental navigation #42 three dimensional dental planning #43 3D dental planning #44 surgical guide #45 surgical stent #46 surgical template #47 MeSH descriptor: [Dimensional Measurement Accuracy] explode all trees #48 accuracy #49 computer-guided accuracy #50 computer-guided precision #51 #1 OR #2 OR #3 OR #4 OR #5 OR #6 OR #7 OR #8 OR #9 OR #10 OR #11 OR #12 OR #13 #52 #14 OR #15 OR #16 OR #17 OR #18 OR #19 OR #20 OR #21 OR #22 OR #23 OR #24 OR #25 #53 #26 OR #27 OR #28 OR #29 OR #30 OR #31 OR #32 OR #33 OR #34 OR #35 OR #36 OR #37 OR #38 OR #39 OR #40 OR #41 OR #42 OR #43 OR #44 OR #45 OR #46 #54 #47 OR #48 OR #49 OR #50 #55 #51 AND #52 AND #53 AND #54 [Custom year range: 2013–2023; Language: English]	61

* Truncation, P: population, I: intervention, C: comparator, O: outcome.

**Table 3:** Demographic characteristics and baseline parameter description of the included studies.

Author, Year, and Country	*n* (Resin Models)	*n* (Implants)	Location of Implant (FDI Two-Digit System)	Implant System	Implant Diameter and Length	Planning Software	Navigation Software	Intervention (Robot System Implant)	Surgical Robot Details	Comparator (Type of Surgery)
Chen et al., 2023 [13], China	*n* = 10 (5 per group) (maxilla and mandible)	*n* = 20 (10 per group)	Partially edentulous maxilla: #12, #14, #16, #21 Partially edentulous mandible: #36, #37, #43, #45, #46	Nobel Parallel CC	#12, #43: 3.5 mm × 8 mm #14, #21, #36, #45: 4.3 mm × 10 mm #37, #45, #46: 4.3 mm × 11.5 mm #16: 5 mm × 10 mm	Cycad DHC-DI, version: V2 (Hangzhou Jianjia Robot Company, Hangzhou, China)	DHC-D12, Digital-health Care Co., Ltd. (Suzhou, China)	THETA robotic dental implant system (Hangzhou Jianjia Robot Company, Hangzhou, China)	Semi-automatic integrated implant surgical robot system Wrist joint rotation range = 360 degree Robot arm moved by the operator using a teaching button to bring near planned position	DCAIS (Yizhimei computer-assisted dynamic navigation system)
Tao et al., 2022 [21], China	*n* = 40 (20 per group) (10 maxilla and 10 mandible per group)	*n* = 480 (240 per group)	Edentulous maxilla: #11, #13, #14, #17, #22, #25, #26 Mandible: #31, #33, #34, #37, #42, #45, #46 Partially edentulous Maxilla (#14, #16, #21, #25, #27) Mandible (#34, #36, #41, #44, #47)	Course Material SP; Institute Straumann AG, Switzerland	4.1 mm × 10 mm	Dental-Helper planning software V1.0.0 (Shanghai, China)	BeiDou-SNS Navigation system V1.0.0 (China)	Hybrid Robotic System for Dental Implant Surgery (HRS-DIS, Shanghai, China)	Prototype DOF: 11 degrees (Serial manipulator (5 DOF), Stewart manipulator (6 DOF)) Human–robot interactive dragging system	DCAIS
Cao et al., 2020 [31], China	*n* = 4 (3 RI group, 1 DI group) (cranio-maxillofacial phantom)	*n* = 16 (12 RI group, 4 SI group)	Cranio-maxillofacial phantom with completely edentulous maxilla	N/M	N/M	CAPPOIS (in-house software)	NDI, Northern Digital Inc.	UR robot (Universal Robots, Odense, Denmark)	DOF: 6 degrees Automatic surgical robot system	DCAIS
Chen et al., 2023 [38], China	*n* = 40 (20 per group) (maxilla)	*n* = 80 (40 per group)	Partially edentulous maxilla: #21, #23 Designated as fresh extraction site: #21 Healed site: #23	Institute Straumann AG (Switzerland)	4.1 × 10 mm	N/M	N/M	Remebot, Beijing Baihui Weikang Technology Co., Ltd.	Task autonomous system Robot arm moved manually by the operator and brought near the planned position	DCAIS
Jin et al., 2022 [39], Korea	*n* = 14 (4 RI group, 10 SI group) (mandible)	*n* = 14 (4 RI group, 10 SI group)	Partially edentulous mandible: #43, #44, #45	TSIII; OSSTEM IMPLANT Co. Ltd., (Seoul, Republic of Korea)	4.5 × 10 mm	RI: MI2RL SI: Implant Studio (3shape A/S, Copenhagen, Denmark)	N/M	Robot arm (Puloon, Seoul, Republic of Korea)	Robot arm: working range = 850 mm; DOF = 6 degree Robot operating software (MI2RL, Seoul, Korea) Position reliability: ±0.1 mm Optical tracker frame rate: 250 Hz	SCAIS (Using 3D printed clear resin guide)

N/M: not mentioned, DCAIS: dynamic computer-assisted implant surgery, SCAIS: static computer-assisted implant surgery, #:tooth number as per the FDI two-digit system, DOF: degree of freedom.

**Table 4:** Details of accuracy data of the included studies.

Author and Year	Accuracy Evaluation Software	Angle Deviation (Degrees) (Mean ± SD)	Coronal Deviation/Entry Deviation/Deviation at Platform (mm) (Mean ± SD)	Coronal Depth Deviation/Depth Deviation at Implant Platform (mm) (Mean ± SD)	Lateral Coronal Deviation/ Linear Lateral Deviation at Platform (mm) (Mean ± SD)	Apical Deviation/Exit Deviation/Deviation at Apex (mm) (Mean ± SD)	Apical Depth Deviation/Depth Deviation at Implant Platform Apex (mm) (Mean ± SD)	Lateral Apical Deviation/Linear Lateral Deviation at Apex (mm) (Mean ± SD)	Authors Suggestions/Conclusions
Chen et al., 2023 [13]	Open-source software 3D Slicer (Version 4.13, Harvard, Boston, Massachusetts, USA)	RS: 1.08 ± 0.66 DCAIS: 2.32 ± 0.71	RS: 0.58 ± 0.31 DCAIS: 0.73 ± 0.20	N/M	N/M	RS: 0.69 ± 0.28 DCAIS: 0.86 ± 0.33	N/M	N/M	Accuracy: RS > DCAIS More accurate implant placements with robotics in terms of angular deviations. No significant difference in accuracy in terms of coronal and apical deviations.
Tao et al., 2022 [21]	N/M	Overall: RS: 1 ± 0.48 DCAIS: 2.41 ± 1.42 Edentulous: Maxilla: RS: 0.83 ± 0.46 DCAIS: 2.44 ± 1.34 Mandible: RS: 1.19 ± 0.54 DCAIS: 2.44 ± 1.66 Partially edentulous: Maxilla: RS: 0.92 ± 0.42 DCAIS: 2.2 ± 1.46 Mandible: RS: 1.07 ± 0.4 DCAIS: 2.55 ± 1.15	Overall: RS: 0.83 ± 0.55 DCAIS: 0.96 ± 0.57 Edentulous: Maxilla: RS: 0.74 ± 0.51 DCAIS: 1 ± 0.52 Mandible: RS: 0.95 ± 0.59 DCAIS: 0.88 ± 0.52 Partially edentulous: Maxilla: RS: 0.74 ± 0.5 DCAIS: 1.02 ± 0.68 Mandible: RS: 0.89 ± 0.59 DCAIS: 0.95 ± 0.58	N/M	N/M	Overall: RS: 0.91 ± 0.56 DCAIS: 1.06 ± 0.59 Edentulous: Maxilla: RS: 0.81 ± 0.75 DCAIS: 1.07 ± 0.57 Mandible: RS: 1.05 ± 0.56 DCAIS: 1.07 ± 0.62 Partially edentulous: Maxilla: RS: 0.8 ± 0.5 DCAIS: 1 ± 0.59 Mandible: RS: 0.99 ± 0.59 DCAIS: 1.09 ± 0.6	N/M	N/M	Accuracy: RS > DCAIS (Significant)
Cao et al., 2020 [31]	N/M	RS: 1.52 ± 0.58 DCAIS: 2.07 ± 0.30	RS: 0.79 ± 0.19 DCAIS: 0.96 ± 0.28	N/M	N/M	RS: 1.49 ± 0.48 DCAIS: 2.26 ± 0.32	N/M	N/M	Accuracy: RS > DCAIS
Chen et al., 2023 [38]	N/M	Fresh extraction site RS: 1.94 ± 0.66 DCAIS: 3.44 ± 1.38 Healed site: RS: 1.36 ± 0.54 DCAIS: 1.80 ± 0.70	Fresh extraction site RS: 0.86 ± 0.36 DCAIS: 0.70 ± 0.21 Healed site: RS: 0.46 ± 0.29 DCAIS: 0.70 ± 0.30	Fresh extraction site RS: 0.43 ± 0.32 DCAIS: 0.00 ± 0.37 Healed site: RS: 0.14 ± 0.34 DCAIS: −0.10 ± 0.40	Fresh extraction site RS: 0.65 ± 0.37 DCAIS: 0.57 ± 0.24 Healed site: RS: 0.38 ± 0.23 DCAIS: 0.57 ± 0.30	Fresh extraction site RS: 0.77 ± 0.34 DCAIS: 0.95 ± 0.38 Healed site: RS: 0.56 ± 0.30 DCAIS: 0.85 ± 0.25	Fresh extraction site RS: 0.42 ± 0.32 DCAIS: −0.02 ± 0.37 Healed site: RS: 0.14 ± 0.34 DCAIS: −0.10 ± 0.40	Fresh extraction site RS: 0.56 ± 0.34 DCAIS: 0.75 ± 0.40 Healed site: RS: 0.47 ± 0.25 DCAIS: 0.75 ± 0.29	A) In fresh extraction site: Accuracy (Angular) RS > DCAIS (significant) Accuracy (Coronal) DCAIS > RS (significant) B) In healed sites: Accuracy (angular, coronal, and apical) RS > DCAIS (significant)
Jin et al., 2022 [39]	Three-dimensional metrology software (Geomagic Control X; Ver. 2020.1, 3D Systems Inc., Roch Hill, South Carolina, USA)	RS: 2.38 ± 0.62 SCAIS: 3.16 ± 2.36	RS: 0.61 ± 0.29 SCAIS: 0.49 ± 0.39	N/M	N/M	RS: 0.50 ± 0.14 SCAIS: 0.72 ± 0.34	RS: 0.17 ± 0.12 SCAIS: 0.15 ± 0.11	N/M	Accuracy: RS = SCAIS (No significant difference)

Note: SD: standard deviation, N/M: not mentioned, RS: robotic system implant surgery; DCAIS: dynamic computer-assisted implant surgery; SCAIS: static computer-assisted implant surgery.

**Table 5:** Quality analysis of the included studies.

Study →			Chen et al., 2023 [13]	Tao et al., 2022 [21]	Cao et al., 2020 [31]	Chen et al., 2023 [38]	Jin et al., 2022 [39]
Section ↓	Item						
Abstract	1	Structured summary	Y	Y	N	Y	Y
Introduction	2a	Specific background	Y	Y	Y	Y	Y
	2b	Specific objectives	Y	Y	Y	Y	Y
Methods	3	Intervention	Y	Y	Y	Y	Y
	4	Outcomes	Y	Y	Y	Y	Y
	5	Sample size	Y	N	N	Y	N
	6	Method for random allocation	N	N	N	Y	N
	7	Allocation concealment mechanism	N	N	N	N	N
	8	Random allocation Implementation	N	N	N	N	N
	9	Blinding	Y	Y	N	Y	N
	10	Outcome comparison methods	Y	Y	N	Y	Y
Results	11	Outcomes and estimation	Y	Y	N	Y	Y
Others	12	Study limitations	Y	Y	Y	Y	Y
	13	Funding source	Y	Y	Y	Y	Y
	14	Availability of full study protocol	Y	N	N	Y	N

Y = Yes; N = No.

## Data Availability

The data that support the findings of this study are available from the corresponding authors upon reasonable request.

## References

[1] Duong H.Y., Roccuzzo A., Stähli A., Salvi G.E., Lang N.P., Sculean A. (2022). Oral health-related quality of life of patients rehabilitated with fixed and removable implant-supported dental prostheses. Periodontology.

[2] Miguel J.A., Freitas T.E. (2020). Immediate orthodontic load on dental implants: An option for adult treatment. Dent. Press J. Orthod..

[3] Ottria L., Lauritano D., Andreasi Bassi M., Palmieri A., Candotto V., Tagliabue A., Tettamanti L. (2018). Mechanical, chemical and biological aspects of titanium and titanium alloys in implant dentistry. J. Biol. Regul. Homeost. Agents..

[4] Kernen F., Kramer J., Wanner L., Wismeijer D., Nelson K., Flügge T. (2020). A review of virtual planning software for guided implant surgery—data import and visualization, drill guide design and manufacturing. BMC Oral Health.

[5] Aydemir C.A., Arısan V. (2020). Accuracy of dental implant placement via dynamic navigation or the freehand method: A split-mouth randomized controlled clinical trial. Clin. Oral Implant. Res..

[6] Ma F., Sun F., Wei T., Ma Y. (2022). Comparison of the accuracy of two different dynamic navigation system registration methods for dental implant placement: A retrospective study. Clin. Implant. Dent. Relat. Res..

[7] Krawiec M., Olchowy C., Kubasiewicz-Ross P., Hadzik J., Dominiak M. (2022). Role of implant loading time in the prevention of marginal bone loss after implant-supported restorations: A targeted review. Dent. Med. Probl..

[8] Silva A.S., Martins D., Sá J., Mendes J.M. (2021). Clinical evaluation of the implant survival rate in patients subjected to immediate implant loading protocols. Dent. Med. Probl..

[9] Safi Y., Amid R., Zadbin F., Ahsaie M.G., Mortazavi H. (2021). The occurrence of dental implant malpositioning and related factors: A cross-sectional cone-beam computed tomography survey. Imaging Sci. Dent..

[10] Parra-Tresserra A., Marquès-Guasch J., Ortega-Martínez J., Basilio-Monné J., Hernández-Alfaro F. (2021). Current state of dynamic surgery. A literature review. Medicina Oral Patología Oral y Cirugía Bucal..

[11] Tattan M., Chambrone L., González-Martín O., Avila-Ortiz G. (2020). Static computer-aided, partially guided, and free-handed implant placement: A systematic review and meta-analysis of randomized controlled trials. Clin. Oral Implant. Res..

[12] Varga E., Antal M., Major L., Kiscsatári R., Braunitzer G., Piffkó J. (2020). Guidance means accuracy: A randomized clinical trial on freehand versus guided dental implantation. Clin. Oral Implant. Res..

[13] Chen J., Bai X., Ding Y., Shen L., Sun X., Cao R., Yang F., Wang L. (2023). Comparison the accuracy of a novel implant robot surgery and dynamic navigation system in dental implant surgery: An in vitro pilot study. BMC Oral Health.

[14] Kaewsiri D., Panmekiate S., Subbalekha K., Mattheos N., Pimkhaokham A. (2019). The accuracy of static vs. dynamic computer-assisted implant surgery in single tooth space: A Randomized Control. Trial. Clin. Oral Implant. Res..

[15] Ronsivalle V., Venezia P., Bennici O., D’Antò V., Leonardi R., Giudice A.L. (2023). Accuracy of digital workflow for placing orthodontic miniscrews using generic and licensed open systems. A 3d imaging analysis of non-native.stl files for guided protocols. BMC Oral Health.

[16] Chackartchi T., Romanos G.E., Parkanyi L., Schwarz F., Sculean A. (2022). Reducing errors in guided implant surgery to optimize treatment outcomes. Periodontology.

[17] Panchal N., Mahmood L., Retana A., Emery R. (2019). Dynamic Navigation for Dental Implant Surgery. Oral Maxillofac. Surg. Clin. N. Am..

[18] D’haese J., Ackhurst J., Wismeijer D., De Bruyn H., Tahmaseb A. (2017). Current state of the art of computer-guided implant surgery. Periodontology.

[19] Pyo S.W., Lim Y.J., Koo K.T., Lee J. (2019). Methods Used to Assess the 3D Accuracy of Dental Implant Positions in Computer-Guided Implant Placement: A Review. J. Clin. Med..

[20] Block M.S., Emery R.W. (2016). Static or Dynamic Navigation for Implant Placement-Choosing the Method of Guidance. J. Oral Maxillofac. Surg..

[21] Tao B., Feng Y., Fan X., Zhuang M., Chen X., Wang F., Wu Y. (2022). Accuracy of dental implant surgery using dynamic navigation and robotic systems: An in vitro study. J. Dent..

[22] Yimarj P., Subbalekha K., Dhanesuan K., Siriwatana K., Mattheos N., Pimkhaokham A. (2020). Comparison of the accuracy of implant position for two-implants supported fixed dental prosthesis using static and dynamic computer-assisted implant surgery: A randomized controlled clinical trial. Clin. Implant. Dent. Relat. Res..

[23] Bover-Ramos F., Viña-Almunia J., Cervera-Ballester J., Peñarrocha-Diago M., García-Mira B. (2018). Accuracy of Implant Placement with Computer-Guided Surgery: A Systematic Review and Meta-Analysis Comparing Cadaver, Clinical, and In Vitro Studies. Int. J. Oral Maxillofac. Implants..

[24] Jorba-García A., González-Barnadas A., Camps-Font O., Figueiredo R., Valmaseda-Castellón E. (2021). Accuracy assessment of dynamic computer-aided implant placement: A systematic review and meta-analysis. Clin. Oral Investig..

[25] Schnutenhaus S., Edelmann C., Knipper A., Luthardt R.G. (2021). Accuracy of Dynamic Computer-Assisted Implant Placement: A Systematic Review and Meta-Analysis of Clinical and in Vitro Studies. J. Clin. Med..

[26] Sun T.M., Lan T.H., Pan C.Y., Lee H.E. (2018). Dental implant navigation system guide the surgery future. Kaohsiung J. Med. Sci..

[27] Golob Deeb J., Bencharit S., Carrico C.K., Lukic M., Hawkins D., Rener-Sitar K., Deeb G.R. (2019). Exploring training dental implant placement using computer-guided implant navigation system for predoctoral students: A pilot study. Eur. J. Dent. Educ..

[28] Sun T.M., Lee H.E., Lan T.H. (2019). The influence of dental experience on a dental implant navigation system. BMC Oral Health.

[29] Feng Y., Fan J., Tao B., Wang S., Mo J., Wu Y., Liang Q., Chen X. (2022). An image-guided hybrid robot system for dental implant surgery. Int. J. Comput. Assist. Radiol. Surg..

[30] De Benedictis A., Trezza A., Carai A., Genovese E., Procaccini E., Messina R., Randi F., Cossu S., Esposito G., Palma P. (2017). Robot-assisted procedures in pediatric neurosurgery. Neurosurg. Focus.

[31] Cao Z., Qin C., Fan S., Yu D., Wu Y., Qin J., Chen X. (2020). Pilot study of a surgical robot system for zygomatic implant placement. Med. Eng. Phys..

[32] Cheng K.J., Kan T.S., Liu Y.F., Zhu W.D., Zhu F.D., Wang W.B., Jiang X.F., Dong X.T. (2021). Accuracy of dental implant surgery with robotic position feedback and registration algorithm: An in-vitro study. Comput. Biol. Med..

[33] Kim M.J., Jeong J.Y., Ryu J., Jung S., Park H.J., Oh H.K., Kook M.S. (2022). Accuracy of digital surgical guides for dental implants. Maxillofac. Plast. Reconstr. Surg..

[34] Wu Y., Wang F., Fan S., Chow J.K. (2019). Robotics in Dental Implantology. Oral Maxillofac. Surg. Clin. N. Am..

[35] Bolding S.L., Reebye U.N. (2022). Accuracy of haptic robotic guidance of dental implant surgery for completely edentulous arches. J. Prosthet. Dent..

[36] Haidar Z. (2017). Autonomous Robotics: A fresh Era of Implant Dentistry… is a reality!. J. Oral. Res..

[37] Mozes A., Vaish S., Cole D.P., Anderson R., He W., Salcedo J., McMahan W.C. (2022). Inventors; Neocis Inc., Assignee. Tracking and Guidance Arrangement for a Surgical Robot System and Related Method. United. States patent.

[38] Chen J., Zhuang M., Tao B., Wu Y., Ye L., Wang F. (2023). Accuracy of immediate dental implant placement with task-autonomous robotic system and navigation system: An in vitro study. Clin. Oral Implant. Res..

[39] Jin X., Kim R.J.-Y., Park J.-M., Jung U.-W., Cha J.-K., Shim J.-S., Heo S.-J. (2022). Accuracy of Surgical Robot System Compared to Surgical Guide for Dental Implant Placement: A Pilot Study. J. Implant. Appl. Sci..

[40] Moher D., Shamseer L., Clarke M., Ghersi D., Liberati A., Petticrew M., Shekelle P., Stewart L.A., PRISMA-P Group (2015). Preferred reporting items for systematic review and meta-analysis protocols (PRISMA-P) 2015 statement. Syst. Rev..

[41] Faggion C.M. (2012). Guidelines for reporting pre-clinical in vitro studies on dental materials. J. Evid. Based Dent. Pract..

[42] (2020). Review Manager (RevMan) [Computer Program], Version 5.4.1; The Cochrane Collaboration; Cochrane: London, UK. https://training-cochrane-org.vgharpa.vghtpe.gov.tw/online-learning/core-softwarecochrane-reviews/revman.

[43] Jia S., Wang G., Zhao Y., Wang X. (2023). Accuracy of an autonomous dental implant robotic system versus static guide-assisted implant surgery: A retrospective clinical study. J. Prosthet. Dent..

[44] Mozer P.S. (2020). Accuracy and Deviation Analysis of Static and Robotic Guided Implant Surgery: A Case Study. Int. J. Oral Maxillofac. Implant..

[45] Chen W., Al-Taezi K.A., Chu C.H., Shen Y., Wu J., Cai K., Chen P., Tang C. (2023). Accuracy of dental implant placement with a robotic system in partially edentulous patients: A prospective, single-arm clinical trial. Clin. Oral Implant. Res..

[46] Chen Z., Li J., Sinjab K., Mendonca G., Yu H., Wang H.L. (2018). Accuracy of flapless immediate implant placement in anterior maxilla using computer-assisted versus freehand surgery: A cadaver study. Clin. Oral Implant. Res..

[47] Zhou L.P., Zhang R.J., Sun Y.W., Zhang L., Shen C.L. (2021). Accuracy of Pedicle Screw Placement and Four Other Clinical Outcomes of Robotic Guidance Technique versus Computer-Assisted Navigation in Thoracolumbar Surgery: A Meta-Analysis. World Neurosurg..

[48] Somogyi-Ganss E., Holmes H.I., Jokstad A. (2015). Accuracy of a novel prototype dynamic computer-assisted surgery system. Clin. Oral Implant. Res..

